# Autism Spectrum Disorder with Epilepsy: A Research Protocol for a Clinical and Genetic Study

**DOI:** 10.3390/genes15010061

**Published:** 2023-12-31

**Authors:** Roberto Canitano, Yuri Bozzi

**Affiliations:** 1 Division of Child and Adolescent Neuropsychiatry, University Hospital of Siena, 53100 Siena, Italy; 2 Center for Mind/Brain Sciences (CIMeC), University of Trento, 38068 Rovereto, Italy; yuri.bozzi@unitn.it; 3 CNR Institute of Neuroscience, 56124 Pisa, Italy

**Keywords:** autism spectrum disorder, epilepsy, electroencephalography, childhood and adolescence

## Abstract

Autism spectrum disorder (ASD) is a common neurodevelopmental condition affecting ~1% of people worldwide. Core ASD features present with impaired social communication abilities, repetitive and stereotyped behaviors, and atypical sensory responses and are often associated with a series of comorbidities. Among these, epilepsy is frequently observed. The co-occurrence of ASD and epilepsy is currently thought to result from common abnormal neurodevelopmental pathways, including an imbalanced excitation/inhibition ratio. However, the pathological mechanisms involved in ASD-epilepsy co-morbidity are still largely unknown. Here, we propose a research protocol aiming to investigate electrophysiological and genetic features in subjects with ASD and epilepsy. This study will include a detailed electroencephalographic (EEG) and blood transcriptomic characterization of subjects with ASD with and without epilepsy. The combined approach of EEG and transcriptomic studies in the same subjects will contribute to a novel stratification paradigm of the heterogeneous ASD population based on quantitative gene expression and neurophysiological biomarkers. In addition, our protocol has the potential to indicate new therapeutic options, thus amending the current condition of absence of data and guidelines for the treatment of ASD with epilepsy.

## 1. Overall Scientific Premise

Autism spectrum disorder (ASD) is a prevalent neurodevelopmental condition which affects approximately 1% of the world’s population [1]. Key characteristics include deficits in social communication, stereotyped and repetitive behaviors, and atypical responses to sensory stimuli [2]. Nevertheless, there exists substantial heterogeneity in clinical manifestations, encompassing diverse symptom profiles, severity levels, and variations in intellectual and functional communication abilities. Furthermore, ASD is notably more frequent in males than females, with biological sex emerging as a significant source of variability in its presentation.

The association between ASD and epilepsy is common, with co-occurrence rates varying widely based on the selected population and accompanying predisposing factors [3,4]. Research indicates an approximately 12% prevalence of epilepsy in people with autism, particularly higher in females, while ASD occurs in about 6% of people with epilepsy, with a higher prevalence in males. The most significant factor linked to ASD and epilepsy comorbidity is the presence of intellectual disability (ID); differences are particularly notable in cognitive functions. A meta-analysis revealed that epilepsy coexisted with ASD in about 21% of patients with both ASD and ID, with a lower prevalence of 8% in those without ID [5]. Another meta-analysis focusing on epilepsy occurrence in ASD found a prevalence of about 9% in people over 12 years without ID and of about 24% in those with ID [6]. Currently, the simultaneous presence of ASD and epilepsy is viewed as the outcome of shared abnormal neurodevelopmental pathways, with evidence supporting common mechanisms underlying epilepsy, autism, and ID [7,8].

Clinical characteristics of ASD with epilepsy have been extensively detailed and warrant thorough examination. Epilepsy is associated with older age, lower cognitive and adaptive function, poorer language ability, developmental regression, and more severe ASD symptoms. A lower IQ of epilepsy patients seems to primarily influence the association between epilepsy and ASD. Independent associations were found between epilepsy, older age, and lower cognitive function. Other risk factors (e.g., poor language and developmental regression) showed no association with epilepsy after controlling for IQ [9,10,11].

Two neuropathological findings, minicolumn abnormalities and γ-aminobutyric acid (GABA) neurotransmission, are common to ASD and epilepsy. ASD and epilepsy exhibit abnormalities in inhibitory GABAergic transmission, including reduced expression of GABA receptor subunits. These abnormalities can disturb the excitation/inhibition (E/I) balance, leading to cortical hyperexcitability and an increased risk of seizures. The minicolumn, a radially oriented assembly in the neocortex, contains pyramidal cell arrays surrounded by GABAergic inhibitory interneurons. Reduced cortical minicolumns have been observed in ASD patients [12,13]. The expression of GABBR1A, GABBR1B, and GABBR2 receptor subunits is reduced in the hippocampi of individuals with temporal lobe epilepsy, and animal models indicate a connection between GABA receptor expression and epilepsy [14]. Similarly, individuals with ASD display abnormalities in GABAergic brain systems, including a reduction in GABA_A_ and GABA_B_ receptor subunits in the fronto-parietal cortex compared with controls [15,16,17].

The genetic abnormalities linking autism and epilepsy belong to the broader spectrum of ASD abnormalities. Cytogenetics allowed us to identify maternally inherited duplications of chromosome 15q11-13 as recurrent, considered a significant cause of ASD alongside other rare chromosomal abnormalities. Array-based methods instead allowed us to identify single genes with major effects, like NLGN, NRXN1, and SHANK3. Although collectively accounting for approximately 15% of cases, variants of these and other genes are detected only in about 1–2% of ASD cases [18]. Moreover, these variants have been observed in both subjects with ASD and those with ID [19]. However, a large percentage of ASD patients, with or without epilepsy, do not show any detectable gene defects, necessitating diagnosis based only on clinical grounds. Copy number variants (CNVs) such as microdeletions, microduplications, insertions, and single-gene mutations, have been associated with ASD. Rare variants, infrequently occurring CNVs, are implicated in these conditions, where multiple rare variants with significant functional effects contribute to common diseases. This heterogeneity is evident in both autism and epilepsy, with multiple phenotypes resulting from a number of rare variants. The significance of the search for rare variants is further underscored by the identification of disease genes closely related to ASD, with more than 100 such genes, including SHANK3, CNTNAP2, and NLGN4X, implicated in both ASD and epilepsy [20]; see also https://gene.sfari.org/ (accessed on 30 November 2023).

## 2. Single Gene Disorders Associated with ASD and Epilepsy

Single gene disorders linked to ASD and epilepsy include Fragile X Syndrome, 22q13 Deletion Syndrome/Phelan-McDermid Syndrome, Rett Syndrome, and Tuberous Sclerosis, each manifesting epilepsy at varying rates. The neurobiological bases of seizures in ASD-associated genetic syndromes are intricate and diverse. E/I imbalance is posited as a potential mechanism in these conditions. For instance, Fragile X implicates defects in the GABA_A_ function [21]. Angelman syndrome is also associated with dysfunction in the GABA_A_ receptor, as genes encoding three GABA_A_ receptor subunits lie proximal to the critical Angelman region (i.e., UBE3A). In Rett syndrome, MECP2 mutations reduce the expression of GABRB3, which encodes the GABA_A_ receptor β3 subunit, and DLX5, which controls GABAergic neuron differentiation [22]. The MECP2 alteration in Rett syndrome has broad effects, acting as a transcriptional factor that regulates the expression of various genes, albeit with poorly understood definitive pathways [23,24].

Although syndromic forms of ASD constitute only a small fraction (approximately 1–2%) of the entire ASD spectrum, they share a series of synaptic signaling abnormalities common to epilepsy. ASD-linked single-gene disorders offer a valid opportunity to comprehend the pathogenic mechanisms of ASD, with or without epilepsy. Altered GABAergic and glutamatergic neurotransmission have been detected in several of these genetic syndromes. Fragile X, for example, implicates defects in GABA_A_ function [25], and recent studies propose that mGluR5 dysfunction heightens excitability, leading to secondary impairment of GABAergic transmission [21]. Angelman syndrome is also associated with dysfunction in GABA_A_ receptor function [26]. A cluster of genes coding for three subunits of the GABA_A_ receptor is located close to the critical Angelman region containing UBE3A. Fragile X, Phelan-McDermid, and Tuberous Sclerosis syndromes are also characterized by profound alterations of synaptic function. These single-gene disorders, by definition, have different genetic causes and phenotypes but are all characterized by the fact that abnormal gene function triggers cascade reactions that derange numerous protein pathways. More specifically, they share synaptic signaling defects leading to altered neurotransmission, E/I imbalance, and impaired synaptic plasticity.

The study of multiplex ASD families yielded significant results about the link between ASD and epilepsy. A recent study revealed that the prevalence of epilepsy was about 13% in people with ASD and about 2% in siblings without ASD. The risk of epilepsy in multiplex ASD was significantly associated with ID but not with gender. Moreover, identified genetic or non-genetic risk factors of ASD, such as prematurity and perinatal disturbances, were significantly associated with epilepsy. The epilepsy phenotype is significantly co-segregated within families; therefore, epilepsy in multiplex ASD seems to have substantial genetic features to define a distinct subgroup [27].

## 3. E/I Imbalance in ASD with Epilepsy

**Figure 1:**
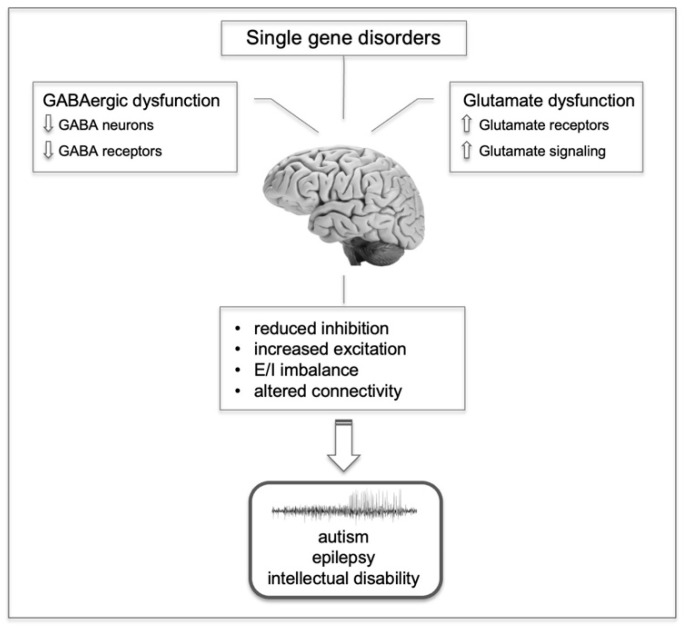
Neural underpinnings of ASD epilepsy comorbidity. The figure schematically shows a multi-factorial model for the expression of ASD-like and epileptic behaviors in rodents. Post-mortem studies in ASD subjects, along with studies on mouse models of ASD-epilepsy [29], indicate that altered GABAergic and glutamatergic signaling may contribute to the co-occurrence of ASD and epilepsy. Single-gene disorders are characterized by mutation in genes coding for synaptic proteins. In addition, increased levels of glutamate receptors and reduced levels of GABA receptors have been detected in ASD brains. As a consequence, E/I imbalance and resulting altered circuit connectivity may ultimately lead to ASD-like behaviors, epilepsy, and ID.

An imbalanced state between excitatory synapses, primarily mediated by excitatory (glutamatergic) and inhibitory (GABAergic) transmission, could impact social cognition during childhood and increase susceptibility to epilepsy [13]. Molecular defects in synaptic structure and function in both ASD and epilepsy involve key proteins such as neuroligins and neurexins, critical for synapse alignment and activation, along with the scaffolding protein SHANK3. Mutations in numerous genes may lead to altered GABAergic interneuron development, serving as a converging point for both ASD and epilepsy [28]. Mutations in genes coding for GABA_A_ receptor subunits have been associated with ASD. Studies revealed that single nucleotide polymorphisms (SNPs) in genes encoding the GABA_A_ receptor subunits α5, β3, and γ3 (respectively GABRA5, GABRB3, and GABRG3, located on chromosome 15q11) are associated with ASD [29]. Mutations and chromosomal rearrangements in Neurexin 1 (NRXN1) have been linked to ASD [30]. Neurexins (NRXNs) are presynaptic proteins that typically bind to neuroligins (NLGNs) located on postsynaptic neurons. NRXNs also bind to GABA_A_ receptors, resulting in decreased GABAergic transmission. Studies on neuroligins in ASD found mutations in NLGN1, 3, and 4X genes [31], and two X-linked neuroligin mutations have been associated with familial ASD [32]. A significant reduction in GABA_A_ receptor α4, α5, β1, and β3 subunits has been observed in ASD brains [15,16,17]. Increased levels of glutamate receptors, likely contributing to hyperexcitability, have been detected in post-mortem brain samples from ASD patients [29]. In agreement with these findings, studies performed on mouse models of ASD–epilepsy revealed an increased excitatory neurotransmission [29]. Figure 1 summarizes these findings.

## 4. Abnormal Connectivity in Autism with Epilepsy

ASD is a dynamic disorder, and the connectivity of the brain undergoes changes with age. Therefore, age and developmental stage need to be considered when assessing functional connectivity in ASD. In a recent study exploring connectivity alterations in the default mode network (DMN) in children, subjects with ASD exhibited both increased and decreased connectivity between the posterior cingulate cortex and the DMN [33]. Connectivity between the posterior cingulate cortex and medial prefrontal cortex was higher in young children with ASD compared with neurotypical controls and then decreased with age. The DMN, active during rest and less so during activity, encompasses brain areas along the midline involved in self-awareness and processing of social emotions—functions impacted in ASD. EEG findings are concordant with MRI findings, showing both over- and under-connectivity in ASD during externally directed, attention-demanding cognitive tasks [34,35].

Epileptiform abnormalities without seizures occur in up to 20–30% of subjects with ASD and epilepsy, but the role of these alterations in the genesis of ASD remains unknown [36]. White matter overgrowth during the first two years of life in ASD is accompanied by arrested or abnormal growth of dendritic trees. Diminished dendritic connections likely play a role in restricting widespread paroxysms, potentially explaining the high occurrence of epileptiform abnormalities without seizures [37]. Corresponding with this hypothesis, structural and functional magnetic resonance imaging (MRI) studies reveal reduced or abnormal connectivity between various brain areas, supporting the notion of underconnectivity between cortical regions. Furthermore, children with stereotypes and aggressive behavior showed a higher incidence of epileptiform abnormalities, which occurred at a significantly lower rate in higher-functioning ASD subjects compared with those with ASD and ID [36].

## 5. Autism with Regression and Epilepsy

Autism with regression and epilepsy occurs in approximately 30% of children with ASD, particularly in those showing an initially normal development and later exhibiting impairments typical of ASD. This disorder involves language loss, typically at the single-word stage, and social withdrawal during the second to third year of age without apparent emotional or environmental factors. In some cases, autistic epileptiform regression occurs, linked with epilepsy. Conditions like Continuous Spikes and Waves during Slow-wave Sleep (CSWSS) and Landau–Kleffner (LKS) syndromes, both rare epileptic encephalopathies, share clinical features such as seizures and regression with autistic features [38,39]. Significantly, the genetic abnormalities identified in these conditions frequently coincide with those linked to ASD or language impairment, underscoring the clinical and genetic convergence of these disorders. While certain studies indicate a higher occurrence of epilepsy and epileptiform abnormalities in children experiencing both ASD and regression, others fail to identify significant associations [40,41].

## 6. Treatment of ASD with Epilepsy

The unpredictability of seizures in ASD individuals underscores the need for consideration by clinicians and family members. A comprehensive set of assistance measures, including emergency and prophylactic norms similar to those used in general care for children with epilepsy, should be explained to the family. The causes and clinical course of seizures in ASD individuals are elusive and not determinative in treatment decisions or clinical consultations. Seizure frequency in young people with ASD is extremely heterogeneous, ranging from a rare occurrence to intractable epilepsy [42,43]. Therefore, a case-by-case approach is essential for management and outcome prediction. Epilepsy poses an additional challenge for individuals with ASD, who already face varying degrees of adaptive difficulties [44]. In cases of seizures occurring in individuals with ASD, the prescription of antiepileptic drugs (AEDs) should align with clinical characteristics and adhere to the prevailing ILAE guidelines for epilepsy [45,46]. Close supervision is necessary to ensure treatment compliance and patient safety. While clinical trials of novel treatments are currently lacking for individuals with ASD and epilepsy, valproate, lamotrigine, and levetiracetam are still considered the most effective and associated with a low rate of cognitive and neurological side effects [47]. Valproate, specifically, has been observed to positively impact behavior, showing improvements in core ASD symptoms as reported in case studies and open-label trials, with minimal adverse effects on cognition [48,49,50,51,52,53,54]. Lamotrigine is another broad-spectrum AED that has been employed to reduce core ASD symptoms, but more data are needed [55]. Levetiracetam, a relatively broad-spectrum AED, has a low incidence of serious adverse effects, with behavioral issues being the most common [56]. Transcranial magnetic stimulation (TMS) is an emerging alternative therapy for certain patients with refractory epilepsy and/or those who do not respond to pharmacotherapy [57]. AEDs have also been used to treat common epileptiform abnormalities and other behavioral symptoms, such as irritability, co-occurring in ASD. However, results from a meta-analysis of seven studies did not show significant differences between medication and placebo [58]. The limitations in power and variations in medication usage across these studies restrict definitive conclusions, underscoring the need for additional research. It is crucial to note that consistent evidence supports the modification of children’s development via early interventions.

Current research shows that children receiving a developmentally based intervention for ASD significantly improve their IQ, adaptive behavior, and overall ASD symptoms. Conversely, untreated groups exhibit more significant developmental delays, including reduced adaptive behavior. The connection between ASD and epilepsy is currently understood as the overlap of shared neurodevelopmental pathways, replacing the previous notion of two distinct, coexisting major disorders. The identification of an increasing number of genes relevant to both ASD and epilepsy strengthens the likelihood of a neurodevelopmental association. Altered synaptic plasticity during early development, arising from early seizures or genetic variations, could potentially act as a shared mechanism influencing the development of both autism and epilepsy. The involvement of copy number variants (CNVs) and structural genomic alterations in strengthening this developmental link implies a potential “double-hit” mechanism. However, it is worth noting that the pathogenic mechanism might also involve multiple genetic hits, especially in the context of a disorder (ASD with epilepsy) that is increasingly seen as polygenic in its origin. Indeed, in terms of its genetic etiology, the autism spectrum includes both rare (Mendelian monogenic) and common (multiple, polygenic) variants. While the study of the effects of rare mutations greatly contributed to unraveling the neurobiological underpinnings of ASD and epilepsy comorbidity, these mutations contribute to a very small percentage of cases (<5%). In the near future, increased accessibility of whole genome/whole exome technologies will certainly guarantee a better understanding of the polygenic causes of ASD and epilepsy. Moreover, the disruption of inhibitory neurotransmission and the consequent E/I imbalance in the developing brain likely contribute to both ASD and epilepsy. Comprehensive endeavors will be necessary to identify the most effective interventions for enhancing overall outcomes in children affected by both disorders.

## 7. Research Design

Here, we propose a research protocol for a clinical and genetic study on ASD and epilepsy, with the aim to collect relevant data on this poorly understood condition and fill the current knowledge gap. The following paragraph describes the proposed protocol.

*Patient recruitment criteria.* Children with ASD and epilepsy will be consecutively enrolled. Diagnosis will be confirmed according to DSMV criteria for ASD and epilepsy by ILAE diagnostic criteria. Participants will be children aged 5–15 years with ASD and epilepsy (*n* = 20, ASD + EPI group). An equal number of girls and boys will be enrolled in order to investigate the possible association with gender prevalence of this association. IQ scores of all participants will be detected using the Wechsler Abbreviated Scale of Intelligence [59], and the absence of neurological conditions or co-occurring neurodevelopmental/psychiatric conditions are inclusion criteria. Participants receiving AED medication will be asked to take a short drop/wash out period, according to the pharmacokinetics of the drug and clinical situation, e.g., evidence of seizures in the last year. Typically developing children of the same age (*n* = 20, TDC group) and without neurodevelopmental or psychiatric diagnoses will be recruited from local schools and forums. All control participants will be screened for subclinical symptoms using the Strengths and Difficulties Questionnaire (SDQ) [60]. The age range of 5–15 was chosen because it allows for stable estimates of diagnosis and IQ, where there is evidence that the ASD phenotype may change over time (e.g., seizure occurrence, regression, upcoming emotional disorders, etc.). However, it is worth noting that the choice of this discrete age range might be limiting the power of the study; indeed, ASD manifestations vary over the course of one’s lifetime, with symptoms beginning around 2–3 years of age and fluctuating throughout one’s life. We propose using a comprehensive and standardized assessment battery to ensure reproducibility. Our clinical team has undergone the requisite training and ongoing reliability assessments to administer these instruments for rigor in data collection.

*ASD diagnostic and social domains.* The ADOS-2 has been validated in distinguishing ASD from other developmental disabilities; we will use it to characterize the nature of core ASD symptomatology in our sample [61]. The ADI-R [62] will be used to collect information about the ASD profile in participants with mental ages above two years old. We will also use the Social Orienting Task, wherein the presence, latency, and duration of orienting toward a series of four social and four non-social stimuli will be measured by trained investigators via a one-way mirror. The use of video and inter-rater reliability will be coded for 20% of assessments in each group. Participants with ASD will meet gold standard diagnostic criteria for ASD, confirmed by the Autism Diagnostic Observation Schedule—2nd Edition (ADOS-2) [61] and the Autism Diagnostic Interview-Revised (ADI-R) [62]. Language testing will include standardized measures reliant on caregiver reports and objective, standardized assessments. Specifically, caregivers will report on a child’s expressive and receptive verbal and non-verbal communication repertoire on the MacArthur-Bates Communication Development Inventory [63], which is a valid assessment of young children’s language [64,65]. Although not normed in children above 30 months of age, preliminary data collected on this measure suggest that raw scores on the MCDI are useful in capturing the range of language functioning also in the minimally verbal and intellectually disabled sample. The MSEL and Vineland will provide standardized assessments of receptive and expressive language by direct assessment and parent interview, respectively. The repetitive behavior domain will be evaluated using the RBS-R [66] and the ABC Motor Stereotypies subscale. Sensory sensitivity will be measured using the SSP [67] and the SAND [68]. The SSP is a widely used caregiver questionnaire with thirty-eight items investigating sensory experiences during daily life. Behavioral responses are rated by a trained examiner on an algorithm measuring specific sensory hyper/hypo-reactivity and seeking behaviors across domains. The corresponding caregiver interview consists of thirty-six items and reports whether a given sensory behavior is present or absent. Cognitive assessments will include the MSEL [69], which will provide information on language, visual reasoning, and fine motor skills. The MSEL is validated in children from birth to 68 months but is nevertheless relevant to intellectually disabled and minimally verbal children at older ages [70]. Age equivalents (AE) and ratio nonverbal IQ (NVDQ) will be computed for comparisons between PMS and ASD. NVDQ will be derived by dividing the mean AE on the Visual Reception and Fine Motor scales of the MSEL by the child’s chronological age and then multiplying by 100. Behavioral assessments will be performed using structured psychiatric evaluations to collect information about psychiatric comorbidity. We will use the Aberrant Behavior Checklist (ABC) [71] as a phenotyping measure and clinical outcome measure. The ABC is a parent checklist that has been validated in children with ASD and ID and is frequently used as an outcome measure in ASD clinical trials. Table 1 summarizes the clinical phenotyping measures that will be used in the study.

*Epilepsy diagnosis and workup.* According to the current clinical definition, epilepsy is a disease of the brain characterized by any of the following conditions: (1) at least two unprovoked (or reflex) seizures occurring > 24 h apart; (2) one unprovoked (or reflex) seizure and a probability of further seizures similar to the general recurrence risk (at least 60%) after two unprovoked seizures, occurring over the next 10 years; or (3) diagnosis of an epilepsy syndrome. For patients who will meet the criteria for ASD and epilepsy, medical records for demographics, age of nonfebrile seizure onset, age of ASD diagnosis, and language (verbal, limited speech, and nonverbal) will be collected. The most recent MRI findings, genetic testing, and all EEG reports will be collected. Seizures will be classified as focal or generalized, on semiology and electrographic features. Epilepsy diagnoses will be based on ictal and interictal EEG features and medical reports. EEGs will be analyzed for ictal/interictal features and background rhythmic activity. The primary inclusion criterion for epilepsy will be that each subject had an epilepsy diagnosis and at least one seizure in the year before the study.

*EEG acquisition and processing*. Participants will be allowed to explore the environment where registration will be carried out. To obtain the collaboration of young patients and TD children, a favorite movie will be played, and some reinforcers like candies and snacks will be given. After the child successfully wears the training net, the process will be repeated with the actual EEG net. During the EEG recording, the caregiver will remain with the child to provide behavioral support. The caregiver will be given the option to sit with the child or remain behind a curtain. However, in case of a child’s agitation, the recording will be paused before continuing so that the child might recover his/her well-being [72,73].

EEG will be recorded continuously from 62 Ag/AgCl active (actiCAP) scalp electrodes placed according to the extended 10–20 system using a Galileo NT Line—EEG.NET. The Microsoft SQL-based NeuroWorks database will organize the management of patient, study, and laboratory data. The data will be referenced online to electrode FCz and sampled at 500 Hz. Electrode impedances will be kept below 10 kΩ. EEG data will be processed with The Galileo NT Line suite that will allow the process of collecting, monitoring, trending and managing data for EEG recording. Automated artifact detection will be used to exclude any epochs with artifacts, defined as those with amplitudes exceeding ±90 μv or a peak-to-peak amplitude change of 200 μv. Clean epochs will be subjected to Fast Fourier Transform (FFT) with a 10% Hanning window taper to obtain absolute spectral power in the delta (0.5–3.5 Hz), theta (4–8 Hz), α (8–12 Hz) and β (12–20 Hz) frequency bands. Absolute power in each band/condition will be averaged over clusters of electrodes at frontal (F1–F8, Fz), central (C1–C6, Cz), parietal (P3–4, P7–8, Pz), and occipital (O1–2, Oz) scalp locations for analysis. Power data will be log-transformed to approximate a normal distribution prior to statistical analysis. The number of epochs included in the analysis will be equal between groups. The children included in the final analysis should have at least 60 s of artifact-free data for analysis if a shorter time will not be considered in the study.

*Transcriptome analysis.* We will use RNA sequencing (RNAseq) to investigate the transcriptome of blood samples from ASD + EPI and TDC controls (*n* = 20 per group, 40 samples in total). We chose this strategy instead of performing a genetic/genomic screening of ASD + EPI subjects vs. TDC controls to allow for a better mechanistic understanding of this disorder. Several studies carried out on neurodevelopmental and neurodegenerative disorders clearly indicate that even blood transcriptome analysis may provide insightful indications of mechanistic alterations occurring in the brains of affected individuals [74]. Analyses will be performed with an Illumina Hi Seq2500 platform. Data analysis of RNAseq experiments (e.g., alignment, data quality assessment, differential expression, gene enrichment, and ontology analyses) will be performed using R software and Bioconductor packages, as well as other open-source software tools. Weighted gene coexpression network (WGCNA) and cell deconvolution analyses will then be used to decipher pathways and cell types varying among the different experimental groups. This approach has already been successfully used to identify changes in peripheral blood immune cell composition from transcriptome signatures of ASD subjects [75,76].

*Statistics.* To assess the profile of resting-state EEG power in each participant group, we will use ANOVA to compare power in each frequency band between the two groups (ASD and epilepsy, TDC). A separate model will be used for each frequency band (delta, theta, α, β). All models will include an electrode cluster (frontal, central, parietal, occipital) as a within-subjects factor. Significant main effects of group and cluster, and interactions between these factors, will be further investigated using planned pairwise contrasts between pairs of groups/clusters with Bonferroni correction applied to control for multiple comparisons. A separate model will be used for power in each of the four frequency bands. IQ and age will be included as covariates in all models, given the known effects of these variables on EEG power [77,78]. We will conduct a dimensional analysis to investigate how symptoms of ASD and epilepsy will be associated with resting-state power in the whole sample. Pearson correlation coefficients will be computed between SCQ scores (ASD symptoms) and resting-state power values.

## 8. Expected Results

An in-depth phenotyping, including transcriptomic correlates of clinical characteristics and an accurate description of epilepsy and electroencephalogram in the study cohort, would be the primary expected results. Children with ASD are expected to show reduced theta and α power compared with children without ASD, but the coexistence of epilepsy would be a new variable that might modify this picture and provide different results. The scope of this study is to obtain preliminary results to inform the study of a larger population of children with ASD and epilepsy. Moreover, children with more severe ASD showed a greater reduction in α power, and the results of this study might provide hints of α power variations in children with ASD and epilepsy. Changes in resting-state theta activity exhibit more diversity in ASD compared to atypical α oscillations. The variations expected in theta activity in children with ASD and epilepsy will be detected and then evaluated in light of the possible increase or reduction in theta activity; reduced theta activity was linked to increased ASD symptoms in previous studies and will be tested in the current study. Children with severe ASD symptoms would have a lower delta, theta, and α power, as shown by Shephard et al. [79]. The coexistence of epilepsy is an unknown variable as to power outcome in ASD, which will be evaluated in the current study, providing new insights into the neurophysiology of ASD and epilepsy.

Secondly, transcriptome analysis has the potential to unravel the genetic profile of the complex association between ASD and epilepsy. More specifically, gene ontology, WGCNA, and cell deconvolution analyses will allow us to identify gene regulatory pathways specifically affected in ASD + EPI patients. Taken together, clinical and transcriptomic studies will hopefully contribute to suggesting new therapeutic options, thus amending the current condition of absence of data and guidelines for treatment for ASD with epilepsy.

## 9. Contribution and Innovation to the Field

To date, advancements in ASD and epilepsy intervention have primarily depended on approaches loosely tied to epilepsy treatment, regardless of ASD connection. This involved the use of heterogeneous cohorts of patients with diverse origins of the disorder, resulting in broad intervention strategies and mixed results. Recently, genetic discoveries and model systems have clarified the neurobiology of various ASD subtypes, and it is anticipated that this understanding will extend to ASD with epilepsy. This, in turn, opens avenues for the development of biomarkers and innovative therapeutics. This proposal is innovative, in our opinion, as it has the potential to shed light on a condition that bears multiple disabilities, and so complicates the correct management and treatment. First, this study will leverage existing work in animal models with ASD and epilepsy and include electrophysiological studies that would elucidate the characteristics of children with both conditions. Second, we have the opportunity to thoroughly describe the clinical phenotype that characterizes this population. Treatment will be based on the individual’s different clinical, instrumental, and genetic profiles, allowing a precision medicine approach. Third, the phenotype and electrophysiological profile identified in autism with epilepsy will be critically relevant to ASD as a whole and for developing novel resting-state EEG biomarkers for ASD with epilepsy. We will use the knowledge gained in ASD to inform the stratification of a subset of ASD with constrained heterogeneity based on biological and neurophysiological markers.

## 10. Limitations of the Proposed Research Design

Broadly, advancing our understanding of the links between genetic variation and the various epilepsies seen in ASD is critically important to more precise phenotypic profiling of the disorder. However, it is important to note that an increasing body of research is demonstrating the link between mosaic mutations and ASD as well as different forms of epilepsy [80,81,82,83]. It is worth noting that the present study is not designed to screen for mosaic mutations in ASD with epilepsy. Given that various disorders characterized by excessive cellular proliferation (e.g., spermatogenesis, cancer, focal epilepsy, and neurodevelopmental disorders such as ASD) share a high degree of genetic similarity, this is a crucially important point and represents a potential limitation of the proposed research design. Related to this, it is important to note that in the proposed study, transcriptomic analyses are limited to blood samples. Given that many types of ASD and epilepsy are linked to germline or somatic mosaicism, the proposed approach will not allow the detection of these forms of the disorders. To address this issue, it would be interesting to genetically screen various tissue samples (e.g., different brain cell types reprogrammed from patient’s cells) and search for links to different forms of epilepsy. This approach would allow us to investigate the link between specific types of epilepsy and mutations associated with E/I imbalance and the link between cell-specific mosaic mutations and the source of the focal epilepsies. This will be essential for advancing the field of precision medicine of neurodevelopmental disorders in the most spatiotemporally detailed manner

## Figures and Tables

**Table 1:** Clinical phenotyping measures.

ASD Diagnostic	Autism Diagnostic Observation Schedule-2 Autism Diagnostic Interview-Revised DSM-5	Direct assessment Caregiver report Direct assessment
Social	ADOS-2 comparison score Social Orienting Aberrant Behavior Checklist—Social Withdrawal Subscale	Direct assessment Direct assessment Caregiver report
Language/Communication	MacArthur-Bates Communication Development Inventory Vineland Adaptive Behavior subscales Mullen Scales for Early Learning subscales	Caregiver report Caregiver report Direct assessment
Repetitive Behavior	Repetitive Behavior Scales-Revised Aberrant Behavior Checklist—Motor Stereotypies Subscale Short Sensory Profile Sensory Assessment for Neurodevelopmental Disorders	Caregiver report Caregiver report Caregiver report Direct assessment
Cognitive	Mullen Scales of Early Learning WISC	Direct assessment Direct Assessment
Adaptive Behavior	Vineland Adaptive Behavior Scales-III	Caregiver report
